# Anti-Oxidant and Pro-Oxidant Effects of Peroxiredoxin 6: A Potential Target in Respiratory Diseases

**DOI:** 10.3390/cells12010181

**Published:** 2023-01-01

**Authors:** Wenhui Jia, Chunling Dong, Bo Li

**Affiliations:** 1 Department of Pulmonary and Critical Care Medicine, Second Hospital, Jilin University, Changchun 130041, China; 2 Jilin Provincial Key Laboratory of Oral Biomedical Engineering, Department of Oral Anatomy and Physiology, Hospital of Stomatology, Jilin University, Changchun 130021, China

**Keywords:** oxidative stress, peroxiredoxin 6, acute lung injury, lung cancer, anti-oxidant, reactive oxygen species

## Abstract

Peroxiredoxin 6 (PRDX6) is widely distributed in several organs, especially the lungs. The role of PRDX6 in oxidative stress is controversial and even contradictory, as indicated by research conducted over the past 20 years. PRDX6 has anti-oxidant or pro-oxidant effects on oxidative stress in different diseases. It can even exhibit both anti-oxidant and pro-oxidant effects in the same disease. These findings are attributed to the fact that PRDX6 is a multifunctional enzyme. The peroxidase and phospholipase A2 activity of PRDX6 is closely related to its anti-oxidant and pro-oxidant effects, which leads to the conflicting regulatory effects of PRDX6 on oxidative stress in respiratory diseases. Moreover, PRDX6 interacts with multiple redox signaling pathways to interfere with cell proliferation and apoptosis. PRDX6 has become a new target in respiratory disease research due to its important regulatory role in oxidative stress. In this paper, the role of PRDX6 in oxidative stress in respiratory diseases and the research progress in targeting PRDX6 are reviewed.

## 1. PRDX6 Introduction

### 1.1. Structure of PRDX6

**Figure 1:**
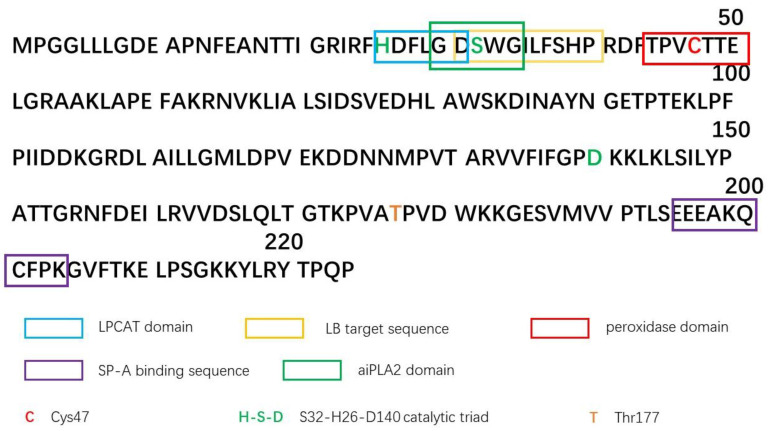
PRDX6 amino acid sequence and functional domain (sequence).

Peroxiredoxins are a highly conserved peroxidase family. Similar to other members (PRDX1–5), PRDX6 has peroxidase activity. Specifically, PRDX6 also has acidic calcium-independent phospholipase (aiPLA2) activity and lysophosphatidylcholine acyltransferase (LPCAT) activity [1,2,3]. The peroxidase activity of PRDX6 is mainly responsible for scavenging peroxides, including H_2_O_2_, short-chain hydroxides, and phospholipid hydroperoxide (PLOOH), while its aiPLA2 activity is involved in the phospholipid cycle, lipid peroxidation repair, and NOX2 activation [1,2]. The activity of these enzymes is relatively independent due to different active centers. The structure of PRDX6 is illustrated in Figure 1, where the main active centers are labeled. Its sequence contains several basic components: (1) Cys47, a peroxidase site [3]; (2) the catalytic triad of H26, S32, and D140, which can hydrolyze phospholipids at the sn-2 site and on which the activity of aiPLA2 depends [4]; (3) the 26HxxxxD31 sequence, which is the LPCAT active domain [5]; and (4) the binding sequence of surfactant protein A (SP-A) and the sequence for targeting lamellar bodies (LBs) [6,7]. Mutations at different sites affect the enzymatic activity of PRDX6 differently. For instance, the C47S mutation retains aiPLA2 activity; the H26A and D130A mutations retain peroxidase activity; the S32A mutation retains partial peroxidase activity and is able to scavenge H_2_O_2_ and short chain hydroxides but not phospholipid hydroperoxides; and the S32T mutation retains all enzymatic activity but is not able to target LBs [8,9,10].

### 1.2. Regulation of PRDX6 Expression and Activity

The highest expression level of PRDX6 is in the lungs. Intracellularly, PRDX6 is localized to the cytoplasm, acidic organelles such as lysosomes, and LBs. PRDX6 has also been found in extracellular fluids such as cerebrospinal fluid and alveolar lavage fluid, but its role remains unclear [11]. The expression of PRDX6 is mainly related to reactive oxygen species (ROS) [12]. Factors that can increase intracellular ROS can up-regulate the expression of PRDX6, such as hyperoxia, oxidants (H_2_O_2_ and paraquat), and ionizing radiation. The PRDX6 promoter region has binding sites for multiple transcription factors, including nuclear factor erythroid 2-related factor 2 (Nrf2), activator protein 1 (AP-1), and nuclear factor kappa-light-chain-enhancer of activated B cells (NF-κB) [13,14,15]. Nrf2 is one of the most important ones. A recent study showed that the regulation of PRDX6 expression by Nrf2 showed completely different results under different oxidative stress intensities. When cells suffer from mild stress, Nrf2 up-regulates the level of PRDX6 to protect cells. On the contrary, when ROS are excessive, the accumulation of Nrf2 and Kruppel-like factor (Klf9) inhibits PRDX6 and leads to apoptosis [16]. In addition, recent studies suggest that some microRNAs are involved in regulating PRDX6 expression. miR-24 and miR-371-3p can inhibit PRDX6 expression and increase intracellular ROS levels [17,18,19]. ROS levels, multiple transcription factors, and microRNAs are collectively involved in the regulation of PRDX6 expression, allowing flexible changes in PRDX6 expression to be performed in response to changing conditions.

The regulation of PRDX6 activity is also affected by many factors. Different subcellular localizations of PRDX6 express different enzymatic activities. In acidic organelles, the aiPLA2 activity of PRDX6 is dominant, while in the cytoplasm (neutral pH), peroxidase activity is predominantly expressed [20]. Binding to different substrates also has an effect. When the substrate is an oxidized phospholipid, PRDX6 is able to express aiPLA2 activity in the cytoplasm; however, when the substrate is a reduced phospholipid, PRDX6 can only bind to it under acidic conditions [21]. The interaction of some proteins with PRDX6 is also involved in regulating its enzymatic activity; for example, SP-A interacts with PRDX6 to inhibit aiPLA2 activity [7]. In addition, the level of ROS not only affects the expression of PRDX6 but also its enzyme activity. A high level of intracellular ROS peroxidizes PRDX6 cysteine (PRDX6-SO_3_H) and increases aiPLA2 activity [22]. The mitogen activated protein kinase (MAPK)-induced phosphorylation of the T177 site of PRDX6 is a crucial factor that can increase the aiPLA2 activity of PRDX6 by more than 10 times [23,24]. Since MAPK is involved in various pathological processes, such as tumors and inflammation, attention should be paid to exploring the role of PRDX6 aiPLA2 activity in these diseases. 

The relatively independent, multiple enzyme activities of PRDX6 allow it to simultaneously play anti-oxidant/pro-oxidant roles to regulate redox balance. 

## 2. Functions of PRDX6

**Figure 2:**
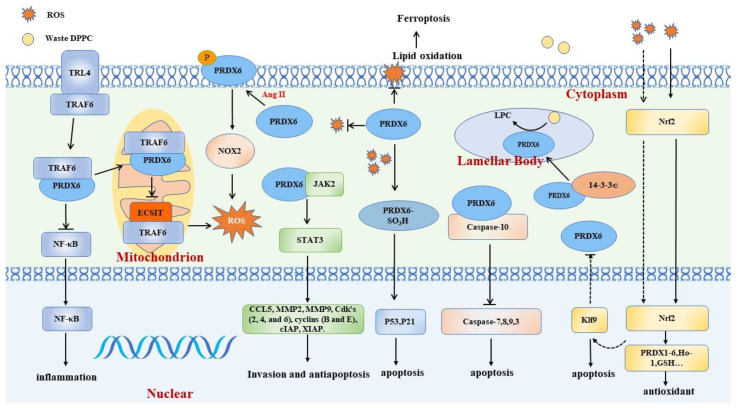
Functions of PRDX6. PRDX6 plays a complex role, with some of the processes in which it is involved including anti-oxidation; pro-oxidation; phospholipid metabolism; and cell proliferation, invasion, and apoptosis regulation. The functions of PRDX6 may be regulated by oxidative stress intensity, cytokines, and other factors.

PRDX6 plays roles in anti-oxidation, pro-oxidation, regulation of cell proliferation/apoptosis, and phospholipid metabolism. This section describes the specific mechanisms of PRDX6, in particular, the action of its enzymatic activity, the interactions between PRDX6 and other proteins, and the signaling pathways in which it is involved (Figure 2).

### 2.1. Anti-Oxidant Effect

PRDX6 exerts its anti-oxidant effect by repairing the peroxidized cell membrane, inhibiting ferroptosis, and reducing the production of mitochondrial ROS (mROS). After oxidative stress induction, phospholipids in the cell membrane are peroxidized to produce PLOOH, resulting in cell damage and even death, so the removal of PLOOH is very important to resist oxidative stress damage [25]. Experimental results show that PRDX6 is the main enzyme in the clearance of PLOOH in the lungs, and the elimination of PRDX6 almost completely abolishes the clearance of PLOOH in the lungs [26,27]. Further studies show that the scavenging of PLOOH requires the joint action of PRDX6 peroxidase and the aiPLA2 enzyme. Furthermore, it was reported that the repair of lipid peroxidation was partial in the lungs and pulmonary microvascular endothelial cells (PMVECs) of mice expressing C47S, H26A or D140A mutant PRDX6 [9,10]. Specifically, the peroxidase activity of PRDX6 can directly reduce PLOOH, while aiPLA2 activity catalyze the hydrolysis of PLOOH to lysophospholipase C (LPC), and then acylation to regenerate phospholipids [5].

Ferroptosis is programmed death dependent on lipid peroxidation; it has been recently described to be closely related to diseases including lung cancer and pulmonary fibrosis [28,29]. Studies confirm that the accumulation of PLOOH is the direct cause of ferroptosis [25]. As the main scavenger of PLOOH in the lungs, PRDX6 can inhibit ferroptosis by clearing PLOOH. In erastin/RSL-3-induced iron death cells, PRDX6 silencing increases the accumulation of PLOOH, and ferroptosis inhibitor ferrostatin-1 can reverse this process [30]. In addition, it was found that the inhibition of ferroptosis by PRDX6 is related to the down-regulation of the iron level by heme oxygenase 1 and the Fenton reaction.

mROS are among the sources of ROS. PRDX6 negatively regulates the production of mROS by combining with TNF (tumor necrosis factor) receptor-associated factor 6 (TRAF6) [31]. Toll-like receptor 4 stimulation can induce the transfer of PRDX6 into the mitochondria. PRDX6 binding with TRAF6 blocks the assembly of the TRAF6-ECSIT complex in mitochondria and the cytoplasm and inhibits the production of mROS and the activation of NF-κB [31].

### 2.2. Pro-Oxidant Effect

PRDX6 has a pro-oxidant effect in some diseases, which depends on the activation of NADPH oxidases 2 (NOX2). NOX2 is a member of the NOX family, and its only function is to generate ROS [32]. NOX2 mainly produces superoxide anions, which are then disproportionated to H_2_O_2_. Six subunits work together to form NOX2. In a static state, gp91phox and gp22phox form a membrane complex, which is spatially separated from other subunits [33]. When activated, membrane complexes and cytoplasmic subunits gather together to produce ROS [34]. The assembly of NOX2 can be initiated by receptor or non-receptor pathways. The former includes angiotensin II, while the latter includes Phorbol-12myristate-13-acetate [35,36]. It was reported that after treatment with agonists, NOX2-mediated ROS increased in the isolated perfused lungs, macrophages, and PMVECs of wild mice. In PRDX6-deficient mice, the activation of NOX2 and ROS production in alveolar epithelial cells were abolished after agonist treatment [36,37]. In line with these results, PRDX6 was reported to affect NOX2-mediated ROS in polymorphonuclear leukocytes (PMN) [38]. The transfer of different mutants into PRDX6-deficient cells further elucidates the enzyme activity needed to activate NOX2. The C47S-PRDX6 mutant is the only mutant that can restore NOX2 activation. Other mutants, including D140A, S32A, H26A, and T177A, are ineffective. This suggests that abolishing the aiPLA2 activity of PRDX6 or stopping the phosphorylation of T177 could prevent the activation of NOX2, giving the same results as the use of aiPLA2 and MAPK inhibitors [36].

Researchers have further investigated the specific mechanism by which PRDX6 activates NOX2. The agonist can phosphorylate PRDX6 in endothelial cells. Subsequently, PRDX6 is transferred to the cell membrane and hydrolyzes the phosphatidylcholine on the membrane into LPC. Then, LPC is converted to lysophosphatidic acid and binds to lysophosphatidic acid receptor 1 to activate RAC, eventually completing the assembly of NOX2 [37]. The activation of NOX2 by PRDX6 is necessary for macrophages, PMVECs, and PMN.

### 2.3. PRDX6 and Cell Proliferation/Apoptosis

PRDX6 has also been confirmed to cross several redox signaling pathways to regulate cell proliferation and apoptosis. The former mainly plays a role in lung cancer, while the latter affects the fate of cells under oxidative stress. 

#### 2.3.1. Proliferation

Yun et al. found that PRDX6 is closely related to the MAPK signaling pathway [39,40]. They reported that in vivo and in vitro, the phosphorylation of MAPK and the DNA binding activity of AP-1 increased in A549/NCI-HH460 cells with high expression of PRDX6. As the growth of tumor cells can affect the phosphorylation of MAPKs, the phenomena observed in the above-mentioned experiment cannot directly indicate that PRDX6 is involved in regulating the MAPK signaling pathway. However, the activation of MAPK may elevate the aiPLA2 activity of PRDX6 and thus promote tumor cell growth. It was observed that the c Jun N terminal kinase (JNK) inhibitor inhibited the enzymatic activity of PRDX6 and the growth of tumor cells, which is consistent with this speculation [39]. MAPKs can regulate the expression and activity of AP-1. There are AP-1 binding sites in the promoter region of PRDX6, and the inhibition of AP-1 can decrease the expression and enzyme activity of PRDX6 [41]. Therefore, the increase in AP-1 expression and activity may further up-regulate the expression of PRDX6 and promote tumor growth and invasion [41].

The team also demonstrated that PRDX6 promotes tumor cell proliferation through JAK2/STAT3 [42]. Compared with wild-type mice, the phosphorylation of JAK2 and STAT3 in the lung cancer tissues of PRDX6-transgenic mice were significantly increased. Immunofluorescence showed increased co-localization and an interaction between PRDX6-transgenic mice and lung cancer patients. After the transfection of different mutants (PRDX6-C47S and PRDX6-S32A), the phosphorylation of JAK2 and STAT3 still increased; therefore, PRDX6 may affect the phosphorylation of JAK2 and STAT3 by directly binding to JAK2 rather than relying on enzyme activity [42]. 

PRDX6 also inhibits the apoptosis of HeLa cells induced by TNF-related apoptosis-inducing ligands (TRAILs) [43]. The decrease in PRDX6 augments the sensitivity of TRAIL-induced apoptosis. PRDX6 inhibits death-inducing signaling complex formation by binding to the death domain of caspase-10 and caspase-8. In vitro, the binding of PRDX6 to caspase-8 and caspase-10 was up-regulated after H_2_O_2_ treatment and decreased after dithiothreitol addition, indicating that the increase in intracellular ROS could weaken the inhibition of PRDX6 of TRAIL-induced apoptosis.

#### 2.3.2. Apoptosis

Recent studies suggest that the changes in intracellular PRDX6 during oxidative stress may affect the fate of cells [16,44]. In lens epithelial cells, under mild oxidative stress, Nrf2 up-regulates PRDX6 to maintain redox balance. In contrast, excessive stress leads to Nrf2 and Klf9 accumulation in the nucleus, the inhibition of PRDX6 expression, and the induction of cell injury [16]. Li et al. confirmed that the Nrf2-Klf9-PRDX6 axis is responsible for the aggravating neurotoxicity of bupivacaine in hyperglycemia patients [45]. The oxidative stress modification of PRDX6 may also induce apoptosis. After treatment with a high concentration of H_2_O_2_ (>100 μm), PRDX6-SO_3_H accumulation occurs in parallel with p53 and P21 protein storage, which may induce cell cycle arrest [22].

### 2.4. PRDX6 and Phospholipid Metabolism

Alveolar surfactant substances synthesized in alveolar type II epithelial cells are secreted into the alveolar lumen to function. “Waste” proteins and phospholipids are endocytosed and transported to LBs for degradation [46]. Part of the degradation product, LPC, is transported to the endoplasmic reticulum to generate new dipalmitoylphosphatidylcholine (DPPC), a process known as DPPC remodeling [47]. PRDX6-deficient mice showed an accumulation of total phospholipids and phosphatidylcholine, a reduction in DPPC degradation and the product LPC, and significantly reduced synthesis of DPPC via the remodeling pathway [48]. Subsequent studies suggested that PRDX6, which degrades DPPC, must be localized in the LBs [48]. The accumulation of phospholipids and reduced DPPC degradation were observed in the lungs of PRDX6-S32T mutant mice, similar to the results in PRDX6-deficient mice. PRDX6 transport to LBs requires binding to chaperone protein 14-3-3∈. PRDX6-S32T mutant mice abolished the binding of PRDX6 to chaperone protein 14-3-3∈, disrupting the PRDX6 targeting of LBs [6]. Thus, PRDX6 localization is necessary for its role in phospholipid metabolism.

## 3. PRDX6 and Respiratory Diseases

**Figure 3:**
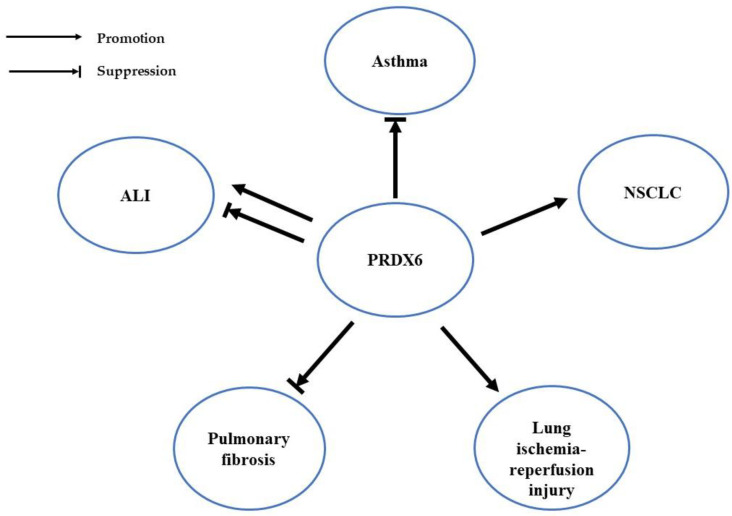
Role of PRDX6 in different respiratory diseases.

PRDX6 plays a role in a variety of respiratory diseases by regulating redox balance, including asthma, non-small-cell lung cancer (NSCLC), acute lung injury (ALI), lung ischemia–reperfusion injury (LIRI), and pulmonary fibrosis (Figure 3), and changes in PRDX6 expression and enzyme activity are often observed in these diseases.

### 3.1. Asthma

Some researchers analyzed changes in serum and lung PRDX6 levels in asthmatics. The results showed that compared with healthy people, the level of PRDX6 in patients with asthma was lower, the peroxidation form of PRDX6 (PRDX6-SO_3_H) was increased, and the ratio of PRDX6-SO_3_H/PRDX6 reflected the severity of asthma [49]. In animal experiments, after exposure to ovalbumin, PRDX6-overexpressing mice had less airway inflammation as well as lowered inflammatory factors and ROS levels in bronchoalveolar lavage fluid [50,51]. However, in PRDX6-null mice, oxidative stress damage factors such as H_2_O_2_, malondialdehyde, and matrix metalloproteinase-9 (MMP-9) increased [52]. Table 1 shows the changes in PRDX6 expression and enzyme activity in asthmatics and in different models.

Shim et al. explored the reason for the decrease in PRDX6 in patients with asthma and attributed it to oxidative stress [53]. Excessive ROS increase PRDX6-SO_3_H and complex post-translational modifications of PRDX6. The accumulation of PRDX6-SO_3_H up-regulates aiPLA2 activity and cytotoxicity, leading to apoptosis [22]. Post-translational modifications include the acetylation of Lys63 sites and the phosphorylation of Ser72, Ser146, Thr177, and Ser186 sites [54]. The modified form is more easily degraded by proteasomes and via autophagy, decreasing PRDX6 in peripheral blood mononuclear cells and bronchial epithelial cells [53]. In addition, modification of these sites may affect the enzyme activity of PRDX6, such as the phosphorylation of T177, but further studies are still needed. 

In general, current studies show that PRDX6 can protect against asthma. Oxidative stress can promote asthma by decreasing the level of PRDX6 and changing its activities.

### 3.2. NSCLC

Many studies report increased expression and activity of PRDX6 in the tumor tissues of patients with NSCLC [55,56,57,58]. Studies suggest that a decrease in the prevalence of malignant lung tumors in Alzheimer’s patients with presenilin-2 mutations is associated with increased PRDX6 degradation [59,60]. In vitro, the viability of A549 cells and NCI-H460 cells increased with PRDX6 expression but decreased after treatment with siRNA [56,61]. The changes in PRDX6 in NSCLC patients and different models are shown in Table 2. Similar phenomena have also been observed in many other tumors, such as colon tumor, cervical tumor, and cholangiocarcinoma, indicating that PRDX6 can promote tumors. On the one hand, PRDX6 maintains cell survival via anti-oxidation. In vitro, siRNA decreases the expression of PRDX6 and inhibits the growth of A549 cells, similar to mercaptosuccinate (a non-specific inhibitor of peroxidase activity), suggesting that PRDX6 promotes the growth of tumor cells through oxidase activity [61]. As the negative regulator of ferroptosis, PRDX6 protects tumor cells by clearing PLOOH. On the other hand, PRDX6 promotes tumor cell proliferation through multiple signaling pathways related to cell proliferation, as mentioned above. An interesting phenomenon was found in previous experiments. PRDX6-C47S mutant transfection resulted in inhibition of A549 cell growth and PRDX6 enzyme activity. This result shows that inhibiting the Cys47 site of PRDX6 can inhibit the occurrence and development of tumors, which provides a possibility for targeting PRDX6 [40,61].

Chemotherapy is an important treatment for lung cancer [64]. Platinum drugs are very common in chemotherapy for lung cancer. A high expression of PRDX6 leads to platinum resistance [65]. Cancer stem cells (CSCs) have stem cell characteristics. Due to various gene mutations, cancer stem cells stop proliferation indefinitely in a specific stage of differentiation and are highly resistant to chemotherapy [66]. CD133 is one of the surface markers of CSCs. In NSCLC patients, the expression levels of PRDX6 and CD133 were reported to be increased and positively correlated. Knocking down PRDX6 with siRNA can reverse the resistance of CSCs to cisplatin and its ability to form globules [57]. A low level of ROS is the key to the resistance of CSCs to chemotherapy [67,68]. Therefore, PRDX6 may enhance the stem-like properties of CSCs and mediate chemotherapy resistance in lung cancer by maintaining a low level of ROS through anti-oxidation.

Radiotherapy is also an important part of treating lung cancer, but radiotherapy can cause various related injuries, such as radiation pneumonia [69]. Therefore, the use of radiation protective agents to reduce radiation damage is an integral part of treatment. Radiation damage depends on the production of ROS in the process of ionizing radiation [70]. PRDX6 is an anti-oxidant enzyme that can be protected by neutralizing ROS and participating in signaling pathways such as TLR4/NF-κB [71,72], so exogenous PRDX6 is a potential radiation protective agent. 

In conclusion, PRDX6 participates in chemotherapy resistance, as well as tumor cell growth and invasion through its anti-oxidant effect and ROS-related signaling pathways. Therefore, targeting PRDX6, thus disturbing redox balance in tumor cells, is a promising line of therapy.

### 3.3. ALI

The primary manifestation of ALI is acute progressive respiratory insufficiency. Many factors can lead to ALI, and pulmonary inflammation is critical. An outbreak of inflammation in the lungs can lead to excessive production of ROS and can damage proteins, lipids, and DNA, promoting lung damage [73]. As an anti-oxidant enzyme, PRDX6 plays a key protective role in ALI. In an ALI model induced with intratracheal instillation of lipopolysaccharide (LPS), PRDX6-deficient mice showed increased ROS levels and inflammatory reaction, as well as more severe pulmonary edema [74]. The increase in ROS production amplified the pulmonary inflammatory response through NF-κB by up-regulating a variety of inflammatory cytokines, including TNF-α and IL-1β. In addition, ROS also up-regulated the levels of MMP-2 and MMP-9 and increased vascular permeability and pulmonary edema [74]. The same results were observed in the ALI model of systemic septicemia induced via cecal ligation and puncture [75]. The results show that PRDX6 plays a protective role in ALI by down-regulating the level of ROS.

As reported above, PRDX6 protects ALI through anti-oxidation. However, with a deepening of the understanding of PRDX6, it was found that PRDX6 also promotes ALI through pro-oxidation [76,77]. The inactivation of the renin–angiotensin system is involved in the pathological process of ALI [78]. Lung injury mediated by elevated levels of angiotensin II was observed in patients and rats with ALI [79,80]. As mentioned above, angiotensin II is the activator of NOX2. Many lung injury models have confirmed that under the action of angiotensin II, PRDX6 is phosphorylated and NOX2 is activated to increase ROS production. In LPS-induced acute lung injury, MJ33 (PRDX6-aiPLA2 activity inhibitor) or PRDX6 inhibitory peptide 2 (PIP-2) could prevent lung injury [81,82,83]. In a systemic septicemia model induced with intraperitoneal injection of LPS, Prdx6-D140A mutant mice showed reduced lung injury and ROS production thanks to the de-activation of NOX2, NF-κB, and nucleotide-binding and oligomerization domain, leucine-rich repeat and pyrin domain-containing 3 (NLRP3) [84]. Table 3 summarizes the effects of different models/treatments on PRDX6 enzyme activity and lung injury. It is worth noting that NOX2 immunity of the host is essential. In neutrophils and macrophages, ROS produced by NOX2 can directly kill pathogenic microorganisms on the one hand and activate many immune signal transduction pathways on the other hand [85]. In cecal-ligation-and-puncture-induced ALI, the number of bacteria in the lungs and peritoneal fluid of mice treated with PIP-2 alone increased, which was reversed with antibiotics [86]. Therefore, antibiotic coverage is necessary when inhibiting PRDX6 aiPLA2 activity or NOX2 in ALI treatment. 

### 3.4. Lung Ischemia–Reperfusion Injury 

LIRI usually refers to the rapid accumulation of ROS and lung tissue injury after pulmonary ischemia–reperfusion. LIRI is a process with a complex mechanism that is closely related to pulmonary embolism and lung transplantation. The increase in ROS production is a key step in mediating lung injury and the release of inflammatory factors [87]. The change in intravascular shear force is a factor in activating NOX2 and producing ROS [88]. In the PMVECs of wild-type mice, the change in shear stress closed the K_ATP_ channel of endothelial cells, activated NOX2, and increased the production of ROS. In contrast, no ROS were produced in mice with NOX2 deletion or K_ATP_ channel knockout. Although the agonists are different, the activation of NOX2 in LIRI also depends on the aiPLA2 activity of Prdx6. In PRDX6-deficient mice, ROS production decreased and oxidative damage was alleviated during pulmonary ischemia. A lung ischemia–reperfusion model of mice treated with MJ33 showed similar results [89,90], as illustrated in Table 4. In clinical practice, LIRI is always a challenge in lung transplantation and a major cause of primary graft dysfunction [91]. Thus, understanding the role of PRDX6 in LIRI is necessary to understand its complex functions. Additionally, the inhibition of the aiPLA2 activity of PRDX6 or NOX2 could be a way to attenuate LIRI during lung ischemia. 

### 3.5. Pulmonary Fibrosis

Hermansky–Pudlak syndrome (HPS) was first reported in 1959 and is mostly inherited in an autosomal recessive fashion. Some patients with HPS develop lethal pulmonary fibrosis accompanied by enlarged LBs filled with surfactant phospholipids [92]. An AP-3 mutant pearl mouse model exhibited LB enlargement and surfactant phospholipid accumulation similar to that in HPS patients. AP-3 is involved in the translocation of proteins to lysosomes or lysosome-associated organelles, and mutations in AP-3 block the transport of PRDX6 to LBs, causing abnormal accumulation of surfactant phospholipids in LBs, similar to that observed in PRDX6-deficient or PRDX6-S32T mutant mice. When the function of AP-3 in alveolar type 2 cells is restored, both the enlarged LBs and the increased surfactant phospholipids are resolved [93].

In addition, Liu et al. found elevated levels of PRDX6 in mouse lungs after silica dust exposure [94]. Elko et al.’s analysis showed that PRDX6 was elevated in patients with NSIP and did not change significantly in patients with IPF [95]. These results suggest that PRDX6 may play a role in some types of pulmonary fibrosis, but the exact mechanism remains unclear.

## 4. Research Progress in Targeting PRDX6

There have been some attempts to target PRDX6 to treat diseases, and some results have been achieved. Thiacremonone inhibits the growth of lung cancer cells by inhibiting the peroxidase of PRDX6 by interacting with the Cys-47 of PRDX6 [62]. Snake venom toxin can bind with the c-Fos of AP-1 to reduce the expression and enzyme activity of PRDX6 and inhibit the growth of A549/NCI-460 cells [41]. Recent studies have described Withangulatin A, the first covalent inhibitor of the PRDX6-Cys47 site, which can effectively inhibit the proliferation of H1975 cells [96]. These results strongly demonstrate the possibility of PRDX6 as a therapeutic target for non-small-cell lung cancer. 

PRDX6 plays a dual role in ALI, and the inhibition of its aiPLA2 activity can significantly reduce lung injury. As SP-A can bind to PRDX6 to inhibit its aiPLA2 activity, Fisher et al. extracted PIP with a similar effect [97]. In various ALI models, PIP-2 treatment reduced lung injury and ALI mortality [81,82,86]. ALI can be prevented or treated by inhibiting the aiPLA2 activity of PRDX6. However, as mentioned above, antibiotic coverage is necessary.

## 5. Conclusions and Perspectives

In summary, as an important regulator of redox balance, PRDX6 plays an important role in maintaining redox balance. We speculate that PRDX6 may also play a dual role in other diseases, not just ALI. For example, the increased level of NOX2 and ROS in the lung tissue of asthmatics may be related to the activation of PRDX6 in neutrophils, which is consistent with the up-regulation of PRDX6-SO_3_H and the phosphorylation of the PRDX6-T177 site in asthmatics. In addition, research on PRDX6-related diseases is still limited. For example, in pulmonary fibrosis disease, although some studies have suggested the engagement of PRDX6, the types of pulmonary fibrosis disease in which PRDX6 is involved and the specific mechanisms remain unclear. Therefore, it is still necessary to further explore the role of PRDX6 in redox imbalance diseases and its duality.

Aside from the above, why PRDX6 plays different roles in different diseases is worth further exploration. Current studies suggest that intracellular ROS levels and some factors can affect the function of PRDX6 and even determine the cell fate. This shows that it is possible to accurately regulate the function and activity of PRDX6, which can provide new ideas and targets for the treatment of many diseases. However, PRDX6 is widely distributed throughout the body and participates in important physiological functions, such as phospholipid circulation. Targeting it may affect the normal physiological activity of the lungs and redox balance in other body parts. While other approaches may compensate for this impact, further assessment is needed to clarify this.

## Figures and Tables

**Table 1:** PRDX6 expression and activity changes in asthmatics and different models.

Disease	Models	Expression	Peroxidase Activity	aiPLA2 Activity	Effect	References
Asthma	Serum of asthmatics	↓	None	None	+	[50]
	PBMCs of asthmatics	↓	None	None	+	[53]
	PRDX6-overexpressing mice	↑	None	None	−	[51]
	PRDX6 knockout mice	↓	↓	↓	+	[52]

↑ up-regulation, ↓ down-regulation, + protective, − detrimental, PBMCs peripheral blood mononuclear cells.

**Table 2:** PRDX6 expression and activity changes in NSCLC models.

Disease	Models	Expression	Peroxidase Activity	aiPLA2 Activity	Effect	References
NSCLC	Human NSCLC patient samples	↑	None	None	+	[55]
	Human NSCLC patient samples	↑	None	None	+	[56]
	Human NSCLC patient samples	↑	None	None	+	[57]
	Human squamous lung cancer patient samples	↑	None	↑	+	[58]
	Thiacremonone-treated A549 cells/NCI-H460 cells	↓	↓	None	−	[62]
	Prdx6 transgenic mice	↑	↑	↑	+	[39]
	Prdx6 transgenic mice	↑	↑	↑	+	[42]
	Pesenilin-2 mutant	↓	↓	↓	−	[58]
	Presenilin-2 knockout mice	↑	↑	↑	+	[60]
	Notch3 knockout	↓	None	None	−	[63]
	PRDX6-siRNA1 injection into the tumorigenesis site	↓	↓	↓	−	[56]
	PRDX6 lentivirus injection into the tumorigenesis site	↑	None	None	+	[56]

↑ up-regulation, ↓ down-regulation, + protective, − detrimental, NSCLC non-small-cell lung cancer.

**Table 3:** PRDX6 expression and activity changes in ALI models.

Disease	Models	Expression	Peroxidase Activity	aiPLA2 Activity	Effect	References
ALI	PRDX6 knockout mice	↓	↓	↓	+	[74]
	PRDX6 knockout mice	↓	↓	↓	+	[75]
	PIP-2 treatment	Unchanged	Unchanged	↓	−	[82]
	PRDX6-D140A mutant mice	Unchanged	Unchanged	↓	−	[84]
	MJ33 treatment	Unchanged	Unchanged	↓	−	[83]

↑ up-regulation, ↓ down-regulation, + protective, − detrimental, PIP-2 PRDX6 inhibitory peptide 2.

**Table 4:** PRDX6 expression and activity changes in LIRI models.

Disease	Models	Expression	Peroxidase Activity	aiPLA2 Activity	Effect	References
Ischemia	PRDX6 knockout mice	↓	↓	↓	−	[89]
	MJ33 treatment	Unchanged	Unchanged	↓	−	[90]

↑ up-regulation, ↓ down-regulation, − detrimental.

## Data Availability

Not applicable.

## References

[1] Fisher A.B. (2018). The phospholipase A(2) activity of peroxiredoxin 6. J. Lipid Res..

[2] Fisher A.B. (2017). Peroxiredoxin 6 in the repair of peroxidized cell membranes and cell signaling. Arch. Biochem. Biophys..

[3] Chen J.W., Dodia C., Feinstein S.I., Jain M.K., Fisher A.B. (2000). 1-Cys peroxiredoxin, a bifunctional enzyme with glutathione peroxidase and phospholipase A2 activities. J. Biol. Chem..

[4] Manevich Y., Reddy K.S., Shuvaeva T., Feinstein S.I., Fisher A.B. (2007). Structure and phospholipase function of peroxiredoxin 6: Identification of the catalytic triad and its role in phospholipid substrate binding. J. Lipid Res..

[5] Fisher A.B., Dodia C., Sorokina E.M., Li H., Zhou S., Raabe T., Feinstein S.I. (2016). A novel lysophosphatidylcholine acyl transferase activity is expressed by peroxiredoxin 6. J. Lipid Res..

[6] Sorokina E.M., Dodia C., Zhou S., Tao J.Q., Gao L., Raabe T., Feinstein S.I., Fisher A.B. (2016). Mutation of Serine 32 to Threonine in Peroxiredoxin 6 Preserves Its Structure and Enzymatic Function but Abolishes Its Trafficking to Lamellar Bodies. J. Biol. Chem..

[7] Krishnaiah S.Y., Dodia C., Sorokina E.M., Li H., Feinstein S.I., Fisher A.B. (2016). Binding sites for interaction of peroxiredoxin 6 with surfactant protein A. Biochim. Biophys. Acta..

[8] Feinstein S.I. (2019). Mouse Models of Genetically Altered Peroxiredoxin 6. Antioxidants.

[9] Li H., Benipal B., Zhou S., Dodia C., Chatterjee S., Tao J.Q., Sorokina E.M., Raabe T., Feinstein S.I., Fisher A.B. (2015). Critical role of peroxiredoxin 6 in the repair of peroxidized cell membranes following oxidative stress. Free Radic. Biol. Med..

[10] Fisher A.B., Vasquez-Medina J.P., Dodia C., Sorokina E.M., Tao J.Q., Feinstein S.I. (2018). Peroxiredoxin 6 phospholipid hydroperoxidase activity in the repair of peroxidized cell membranes. Redox Biol..

[11] Manevich Y., Hutchens S., Halushka P.V., Tew K.D., Townsend D.M., Jauch E.C., Borg K. (2014). Peroxiredoxin VI oxidation in cerebrospinal fluid correlates with traumatic brain injury outcome. Free Radic. Biol. Med..

[12] Kim H.S., Manevich Y., Feinstein S.I., Pak J.H., Ho Y.S., Fisher A.B. (2003). Induction of 1-cys peroxiredoxin expression by oxidative stress in lung epithelial cells. Am. J. Physiol. Lung Cell Mol. Physiol..

[13] Gallagher B.M., Phelan S.A. (2007). Investigating transcriptional regulation of Prdx6 in mouse liver cells. Free Radic. Biol. Med..

[14] Chhunchha B., Kubo E., Singh D.P. (2020). Clock Protein Bmal1 and Nrf2 Cooperatively Control Aging or Oxidative Response and Redox Homeostasis by Regulating Rhythmic Expression of Prdx6. Cells.

[15] Kuda O., Brezinova M., Silhavy J., Landa V., Zidek V., Dodia C., Kreuchwig F., Vrbacky M., Balas L., Durand T. (2018). Nrf2-Mediated Antioxidant Defense and Peroxiredoxin 6 Are Linked to Biosynthesis of Palmitic Acid Ester of 9-Hydroxystearic Acid. Diabetes.

[16] Chhunchha B., Kubo E., Singh D.P. (2022). Switching of Redox Signaling by Prdx6 Expression Decides Cellular Fate by Hormetic Phenomena Involving Nrf2 and Reactive Oxygen Species. Cells.

[17] Soriano-Arroquia A., Gostage J., Xia Q., Bardell D., McCormick R., McCloskey E., Bellantuono I., Clegg P., McDonagh B., Goljanek-Whysall K. (2021). miR-24 and its target gene Prdx6 regulate viability and senescence of myogenic progenitors during aging. Aging Cell.

[18] Sahu N., Stephan J.P., Cruz D.D., Merchant M., Haley B., Bourgon R., Classon M., Settleman J. (2016). Functional screening implicates miR-371-3p and peroxiredoxin 6 in reversible tolerance to cancer drugs. Nat. Commun..

[19] Li Q., Wang N., Wei H., Li C., Wu J., Yang G. (2016). miR-24-3p Regulates Progression of Gastric Mucosal Lesions and Suppresses Proliferation and Invasiveness of N87 Via Peroxiredoxin 6. Dig. Dis. Sci..

[20] Akiba S., Dodia C., Chen X., Fisher A.B. (1998). Characterization of acidic Ca(2+)-independent phospholipase A2 of bovine lung. Comp. Biochem. Physiol. B Biochem. Mol. Biol..

[21] Manevich Y., Shuvaeva T., Dodia C., Kazi A., Feinstein S.I., Fisher A.B. (2009). Binding of peroxiredoxin 6 to substrate determines differential phospholipid hydroperoxide peroxidase and phospholipase A(2) activities. Arch. Biochem. Biophys..

[22] Kim S.Y., Jo H.Y., Kim M.H., Cha Y.Y., Choi S.W., Shim J.H., Kim T.J., Lee K.Y. (2008). H2O2-dependent hyperoxidation of peroxiredoxin 6 (Prdx6) plays a role in cellular toxicity via up-regulation of iPLA2 activity. J. Biol. Chem..

[23] Rahaman H., Zhou S., Dodia C., Feinstein S.I., Huang S., Speicher D., Fisher A.B. (2012). Increased phospholipase A2 activity with phosphorylation of peroxiredoxin 6 requires a conformational change in the protein. Biochemistry.

[24] Wu Y., Feinstein S.I., Manevich Y., Chowdhury I., Pak J.H., Kazi A., Dodia C., Speicher D.W., Fisher A.B. (2009). Mitogen-activated protein kinase-mediated phosphorylation of peroxiredoxin 6 regulates its phospholipase A(2) activity. Biochem. J..

[25] Maiorino M., Conrad M., Ursini F. (2018). GPx4, Lipid Peroxidation, and Cell Death: Discoveries, Rediscoveries, and Open Issues. Antioxid Redox Signal.

[26] Wang Y., Feinstein S.I., Fisher A.B. (2008). Peroxiredoxin 6 as an antioxidant enzyme: Protection of lung alveolar epithelial type II cells from H_2_O_2_-induced oxidative stress. J. Cell Biochem..

[27] Wang Y., Feinstein S.I., Manevich Y., Ho Y.S., Fisher A.B. (2004). Lung injury and mortality with hyperoxia are increased in peroxiredoxin 6 gene-targeted mice. Free Radic. Biol. Med..

[28] Zhang W., Sun Y., Bai L., Zhi L., Yang Y., Zhao Q., Chen C., Qi Y., Gao W., He W. (2021). RBMS1 regulates lung cancer ferroptosis through translational control of SLC7A11. J. Clin. Investig..

[29] Li X., Duan L., Yuan S., Zhuang X., Qiao T., He J. (2019). Ferroptosis inhibitor alleviates Radiation-induced lung fibrosis (RILF) via down-regulation of TGF-β1. J. Inflamm..

[30] Lu B., Chen X.B., Hong Y.C., Zhu H., He Q.J., Yang B., Ying M.D., Cao J. (2019). Identification of PRDX6 as a regulator of ferroptosis. Acta Pharmacol. Sin..

[31] Min Y., Wi S.M., Shin D., Chun E., Lee K.Y. (2017). Peroxiredoxin-6 Negatively Regulates Bactericidal Activity and NF-κB Activity by Interrupting TRAF6-ECSIT Complex. Front. Cell Infect. Microbiol..

[32] Fukai T., Ushio-Fukai M. (2020). Cross-Talk between NADPH Oxidase and Mitochondria: Role in ROS Signaling and Angiogenesis. Cells.

[33] Vermot A., Petit-Härtlein I., Smith S.M.E., Fieschi F. (2021). NADPH Oxidases (NOX): An Overview from Discovery, Molecular Mechanisms to Physiology and Pathology. Antioxidants.

[34] Rastogi R., Geng X., Li F., Ding Y. (2016). NOX Activation by Subunit Interaction and Underlying Mechanisms in Disease. Front. Cell Neurosci..

[35] Birk M., Baum E., Zadeh J.K., Manicam C., Pfeiffer N., Patzak A., Helmstädter J., Steven S., Kuntic M., Daiber A. (2021). Angiotensin II Induces Oxidative Stress and Endothelial Dysfunction in Mouse Ophthalmic Arteries via Involvement of AT1 Receptors and NOX2. Antioxidants.

[36] Chatterjee S., Feinstein S.I., Dodia C., Sorokina E., Lien Y.C., Nguyen S., Debolt K., Speicher D., Fisher A.B. (2011). Peroxiredoxin 6 phosphorylation and subsequent phospholipase A2 activity are required for agonist-mediated activation of NADPH oxidase in mouse pulmonary microvascular endothelium and alveolar macrophages. J. Biol. Chem..

[37] Vázquez-Medina J.P., Dodia C., Weng L., Mesaros C., Blair I.A., Feinstein S.I., Chatterjee S., Fisher A.B. (2016). The phospholipase A2 activity of peroxiredoxin 6 modulates NADPH oxidase 2 activation via lysophosphatidic acid receptor signaling in the pulmonary endothelium and alveolar macrophages. FASEB J..

[38] Ambruso D.R., Ellison M.A., Thurman G.W., Leto T.L. (2012). Peroxiredoxin 6 translocates to the plasma membrane during neutrophil activation and is required for optimal NADPH oxidase activity. Biochim. Biophys. Acta..

[39] Jo M., Yun H.M., Park K.R., Hee Park M., Myoung Kim T., Ho Pak J., Jae Lee S., Moon D.C., Park C.W., Song S. (2013). Lung tumor growth-promoting function of peroxiredoxin 6. Free Radic. Biol. Med..

[40] Yun H.-M., Park K.-R., Lee H.P., Lee D.H., Jo M. (2014). PRDX6 promotes lung tumor progression via its GPx and iPLA2 activities. Free Radic. Biol. Med..

[41] Lee H.L., Park M.H., Son D.J., Song H.S., Kim J.H., Ko S.C., Song M.J., Lee W.H., Yoon J.H., Ham Y.W. (2015). Anti-cancer effect of snake venom toxin through down regulation of AP-1 mediated PRDX6 expression. Oncotarget.

[42] Yun H.M., Park K.R., Park M.H., Kim D.H., Jo M.R., Kim J.Y., Kim E.C., Yoon D.Y., Han S.B., Hong J.T. (2015). PRDX6 promotes tumor development via the JAK2/STAT3 pathway in a urethane-induced lung tumor model. Free Radic. Biol. Med..

[43] Choi H., Chang J.W., Jung Y.K. (2011). Peroxiredoxin 6 interferes with TRAIL-induced death-inducing signaling complex formation by binding to death effector domain caspase. Cell Death Differ..

[44] Chhunchha B., Kubo E., Singh D.P. (2019). Sulforaphane-Induced Klf9/Prdx6 Axis Acts as a Molecular Switch to Control Redox Signaling and Determines Fate of Cells. Cells.

[45] Li H., Weng Y., Lai L., Lei H., Xu S., Zhang Y., Li L. (2021). KLF9 regulates PRDX6 expression in hyperglycemia-aggravated bupivacaine neurotoxicity. Mol. Cell Biochem..

[46] Olmeda B., Martínez-Calle M., Pérez-Gil J. (2017). Pulmonary surfactant metabolism in the alveolar airspace: Biogenesis, extracellular conversions, recycling. Ann. Anat..

[47] Agassandian M., Mallampalli R.K. (2013). Surfactant phospholipid metabolism. Biochim. Biophys. Acta.

[48] Fisher A.B., Dodia C., Feinstein S.I., Ho Y.S. (2005). Altered lung phospholipid metabolism in mice with targeted deletion of lysosomal-type phospholipase A2. J. Lipid Res.

[49] Kwon H.S., Bae Y.J., Moon K.A., Lee Y.S., Lee T., Lee K.Y., Kim T.B., Park C.S., Moon H.B., Cho Y.S. (2012). Hyperoxidized peroxiredoxins in peripheral blood mononuclear cells of asthma patients is associated with asthma severity. Life Sci..

[50] Dong C., Li B., Yang D., Wang G., Wang X., Bai C. (2012). Peroxiredoxin 6-mediated negative regulation of MUC5AC hyper-production and secretion during asthma. Am. J. Respir. Crit. Care Med..

[51] Dong C., Li B., Yang D., Wang G., Wang X., Bai C. (2011). Overexpression of peroxiredoxin 6 protect mice from ovalbumin-induced airway inflammation and hypersecretion of MUC5AC by reducing ROS levels. Eur. Respir. J..

[52] Yang D., Mou Y., Dong C., Jin M., Bai C. (2013). Deletion of peroxiredoxin 6 potentiates OVA-induced asthma epithelial-mesenchymal transition through EGFR pathway. Eur. Respir. J..

[53] Shim H.J., Park S.Y., Kwon H.S., Song W.J., Kim T.B., Moon K.A., Choi J.P., Kim S.J., Cho Y.S. (2020). Oxidative Stress Modulates the Expression Pattern of Peroxiredoxin-6 in Peripheral Blood Mononuclear Cells of Asthmatic Patients and Bronchial Epithelial Cells. Allergy Asthma Immunol. Res..

[54] Jeong J., Kim Y., Kyung Seong J., Lee K.J. (2012). Comprehensive identification of novel post-translational modifications in cellular peroxiredoxin 6. Proteomics.

[55] Zhang X.Z., Xiao Z.F., Li C., Xiao Z.Q., Yang F., Li D.J., Li M.Y., Li F., Chen Z.C. (2009). Triosephosphate isomerase and peroxiredoxin 6, two novel serum markers for human lung squamous cell carcinoma. Cancer Sci..

[56] Li H., Zhang D., Li B., Zhen H., Chen W., Men Q. (2021). PRDX6 Overexpression Promotes Proliferation, Invasion, and Migration of A549 Cells in vitro and in vivo. Cancer Manag. Res..

[57] Nie Y., Huang H., Guo M., Chen J., Wu W., Li W., Xu X., Lin X., Fu W., Yao Y. (2019). Breast Phyllodes Tumors Recruit and Repolarize Tumor-Associated Macrophages via Secreting CCL5 to Promote Malignant Progression, Which Can Be Inhibited by CCR5 Inhibition Therapy. Clin. Cancer Res..

[58] Park M.H., Yun H.M., Hwang C.J., Park S.I., Han S.B., Hwang D.Y., Yoon D.Y., Kim S., Hong J.T. (2017). Presenilin Mutation Suppresses Lung Tumorigenesis via Inhibition of Peroxiredoxin 6 Activity and Expression. Theranostics.

[59] Driver J.A., Beiser A., Au R., Kreger B.E., Splansky G.L., Kurth T., Kiel D.P., Lu K.P., Seshadri S., Wolf P.A. (2012). Inverse association between cancer and Alzheimer′s disease: Results from the Framingham Heart Study. BMJ.

[60] Yun H.M., Park M.H., Kim D.H., Ahn Y.J., Park K.R., Kim T.M., Yun N.Y., Jung Y.S., Hwang D.Y., Yoon D.Y. (2014). Loss of presenilin 2 is associated with increased iPLA2 activity and lung tumor development. Oncogene.

[61] Ho J.N., Lee S.B., Lee S.S., Yoon S.H., Kang G.Y., Hwang S.G., Um H.D. (2010). Phospholipase A2 activity of peroxiredoxin 6 promotes invasion and metastasis of lung cancer cells. Mol. Cancer Ther..

[62] Jo M., Yun H.M., Park K.R., Park M.H., Lee D.H., Cho S.H., Yoo H.S., Lee Y.M., Jeong H.S., Kim Y. (2014). Anti-cancer effect of thiacremonone through down regulation of peroxiredoxin 6. PLoS ONE.

[63] Li Z., Xiao J., Liu M., Cui J., Lian B., Sun Y., Li C. (2022). Notch3 regulates ferroptosis via ROS-induced lipid peroxidation in NSCLC cells. FEBS Open Bio.

[64] Rossi A., Di Maio M. (2016). Platinum-based chemotherapy in advanced non-small-cell lung cancer: Optimal number of treatment cycles. Expert Rev. Anticancer Ther..

[65] Pak J.H., Choi W.H., Lee H.M., Joo W.D., Kim J.H., Kim Y.T., Kim Y.M., Nam J.H. (2011). Peroxiredoxin 6 overexpression attenuates cisplatin-induced apoptosis in human ovarian cancer cells. Cancer Investig..

[66] Najafi M., Farhood B., Mortezaee K. (2019). Cancer stem cells (CSCs) in cancer progression and therapy. J. Cell Physiol..

[67] Choi H.J., Jhe Y.L., Kim J., Lim J.Y., Lee J.E., Shin M.K., Cheong J.H. (2020). FoxM1-dependent and fatty acid oxidation-mediated ROS modulation is a cell-intrinsic drug resistance mechanism in cancer stem-like cells. Redox Biol..

[68] Huang H., Zhang S., Li Y., Liu Z., Mi L., Cai Y., Wang X., Chen L., Ran H., Xiao D. (2021). Suppression of mitochondrial ROS by prohibitin drives glioblastoma progression and therapeutic resistance. Nat. Commun..

[69] Hanania A.N., Mainwaring W., Ghebre Y.T., Hanania N.A., Ludwig M. (2019). Radiation-Induced Lung Injury: Assessment and Management. Chest.

[70] De Ruysscher D., Niedermann G., Burnet N.G., Siva S., Lee A.W.M., Hegi-Johnson F. (2019). Radiotherapy toxicity. Nat. Rev. Dis. Prim..

[71] Sharapov M.G., Glushkova O.V., Parfenyuk S.B., Gudkov S.V., Lunin S.M., Novoselova E.G. (2021). The role of TLR4/NF-κB signaling in the radioprotective effects of exogenous Prdx6. Arch. Biochem. Biophys..

[72] Sharapov M.G., Novoselov V.I., Gudkov S.V. (2019). Radioprotective Role of Peroxiredoxin 6. Antioxidants.

[73] Cen M., Ouyang W., Zhang W., Yang L., Lin X., Dai M., Hu H., Tang H., Liu H., Xia J. (2021). MitoQ protects against hyperpermeability of endothelium barrier in acute lung injury via a Nrf2-dependent mechanism. Redox Biol..

[74] Yang D., Song Y., Wang X., Sun J., Ben Y., An X., Tong L., Bi J., Wang X., Bai C. (2011). Deletion of peroxiredoxin 6 potentiates lipopolysaccharide-induced acute lung injury in mice. Crit. Care Med..

[75] Wang X., An X., Wang X., Hu X., Bi J., Tong L., Yang D., Song Y., Bai C. (2019). Peroxiredoxin 6 knockout aggravates cecal ligation and puncture-induced acute lung injury. Int. Immunopharmacol..

[76] Zhou Y., Zhang C.Y., Duan J.X., Li Q., Yang H.H., Sun C.C., Zhang J., Luo X.Q., Liu S.K. (2020). Vasoactive intestinal peptide suppresses the NLRP3 inflammasome activation in lipopolysaccharide-induced acute lung injury mice and macrophages. Biomed. Pharmacother..

[77] Li D., Cong Z., Yang C., Zhu X. (2020). Inhibition of LPS-induced Nox2 activation by VAS2870 protects alveolar epithelial cells through eliminating ROS and restoring tight junctions. Biochem. Biophys. Res. Commun..

[78] Liu J., Chen Q., Liu S., Yang X., Zhang Y., Huang F. (2018). Sini decoction alleviates E. coli induced acute lung injury in mice via equilibrating ACE-AngII-AT1R and ACE2-Ang-(1-7)-Mas axis. Life Sci..

[79] Wang R., Zagariya A., Ibarra-Sunga O., Gidea C., Ang E., Deshmukh S., Chaudhary G., Baraboutis J., Filippatos G., Uhal B.D. (1999). Angiotensin II induces apoptosis in human and rat alveolar epithelial cells. Am. J. Physiol..

[80] Zhang M., Gao Y., Zhao W., Yu G., Jin F. (2018). ACE-2/ANG1-7 ameliorates ER stress-induced apoptosis in seawater aspiration-induced acute lung injury. Am. J. Physiol. Lung Cell Mol. Physiol..

[81] Fisher A.B., Dodia C., Chatterjee S. (2021). A Peptide Inhibitor of Peroxiredoxin 6 Phospholipase A(2) Activity Significantly Protects against Lung Injury in a Mouse Model of Ventilator Induced Lung Injury (VILI). Antioxidants.

[82] Fisher A.B., Dodia C., Chatterjee S., Feinstein S.I. (2019). A Peptide Inhibitor of NADPH Oxidase (NOX2) Activation Markedly Decreases Mouse Lung Injury and Mortality Following Administration of Lipopolysaccharide (LPS). Int. J. Mol. Sci..

[83] Benipal B., Feinstein S.I., Chatterjee S., Dodia C., Fisher A.B. (2015). Inhibition of the phospholipase A2 activity of peroxiredoxin 6 prevents lung damage with exposure to hyperoxia. Redox Biol..

[84] Vázquez-Medina J.P., Tao J.Q., Patel P., Bannitz-Fernandes R., Dodia C., Sorokina E.M., Feinstein S.I., Chatterjee S., Fisher A.B. (2019). Genetic inactivation of the phospholipase A(2) activity of peroxiredoxin 6 in mice protects against LPS-induced acute lung injury. Am. J. Physiol. Lung Cell Mol. Physiol..

[85] Lam G.Y., Huang J., Brumell J.H. (2010). The many roles of NOX2 NADPH oxidase-derived ROS in immunity. Semin. Immunopathol..

[86] Fisher A.B., Dodia C., Tao J.Q., Feinstein S.I., Chatterjee S. (2021). Inhibition of Peroxiredoxin 6 PLA2 Activity Decreases Oxidative Stress and the Severity of Acute Lung Injury in the Mouse Cecal Ligation and Puncture Model. Antioxidants.

[87] Ferrari R.S., Andrade C.F. (2015). Oxidative Stress and Lung Ischemia-Reperfusion Injury. Oxid. Med. Cell Longev..

[88] Chatterjee S., Nieman G.F., Christie J.D., Fisher A.B. (2014). Shear stress-related mechanosignaling with lung ischemia: Lessons from basic research can inform lung transplantation. Am. J. Physiol. Lung Cell Mol. Physiol..

[89] Chatterjee S., Feinstein S.I., Hong N., DeBolt K. (2007). Paradoxical Response of Endothelial ROS production in Peroxiredoxin 6 null mice to Ischemia. FASEB J..

[90] Lee I., Dodia C., Chatterjee S., Zagorski J., Mesaros C., Blair I.A., Feinstein S.I., Jain M., Fisher A.B. (2013). A novel nontoxic inhibitor of the activation of NADPH oxidase reduces reactive oxygen species production in mouse lung. J. Pharmacol. Exp. Ther..

[91] Chen-Yoshikawa T.F. (2021). Ischemia-Reperfusion Injury in Lung Transplantation. Cells.

[92] Yokoyama T., Gochuico B.R. (2021). Hermansky-Pudlak syndrome pulmonary fibrosis: A rare inherited interstitial lung disease. Eur. Respir. Rev..

[93] Kook S., Wang P., Young L.R., Schwake M., Saftig P., Weng X., Meng Y., Neculai D., Marks M.S., Gonzales L. (2016). Impaired Lysosomal Integral Membrane Protein 2-dependent Peroxiredoxin 6 Delivery to Lamellar Bodies Accounts for Altered Alveolar Phospholipid Content in Adaptor Protein-3-deficient pearl Mice. J. Biol. Chem.

[94] Liu N., Xue L., Guan Y., Li Q.Z., Cao F.Y., Pang S.L., Guan W.J. (2016). Expression of Peroxiredoxins and Pulmonary Surfactant Protein A Induced by Silica in Rat Lung Tissue. Biomed. Environ. Sci..

[95] Elko E.A., Cunniff B., Seward D.J., Chia S.B., Aboushousha R., van de Wetering C., van der Velden J., Manuel A., Shukla A., Heintz N.H. (2019). Peroxiredoxins and Beyond; Redox Systems Regulating Lung Physiology and Disease. Antioxid. Redox Signal..

[96] Chen C., Gong L., Liu X., Zhu T., Zhou W., Kong L., Luo J. (2021). Identification of peroxiredoxin 6 as a direct target of withangulatin A by quantitative chemical proteomics in non-small cell lung cancer. Redox Biol..

[97] Fisher A.B., Dodia C., Feinstein S.I. (2018). A peptide derived from naturally occurring surfactant protein A (SP-A) inhibits the phospholipase A2 (PLA2) activity of peroxiredoxin 6 (Prdx6) and NOX2 activation and prevents mouse lung injury following intratracheal LPS. Free Radic. Biol. Med..

